# Minimal invasive surgery in treatment of liver metastases from colorectal carcinomas: case studies and survival rates

**DOI:** 10.1186/1471-2482-13-S2-S45

**Published:** 2013-10-08

**Authors:** Domenico Loffredo, Alberto Marvaso, Sandro Ceraso, Nicola Angelo Cinelli, Aldo Rocca, Mario Vitale, Michele Rossi, Eugenio Annibale Genovese, Bruno Amato, Mariapia Cinelli

**Affiliations:** 1 Asl Na 2-Hospital A. Rizzoli of Lacco Ameno (Ischia), Naples, Italy; 2 Perfusion Science, University of Naples "Federico II", Naples, Italy; 3 Bioethics, Catholic University "Sacred Heart", Rome, Italy; 4 Department of Clinical Medicine and Surgery, University of Naples "Federico II" Naples, Naples, Italy; 5 Department of Medicine and Surgery, University of Salerno, Baronissi (SA), Italy; 6 Department of Radiology, Sant'Andrea Hospital, University of Rome "Sapienza", Rome, Italy; 7 Department of Radiology, University of Cagliari, Cagliari, Italy; 8 Department of Public Health, University of Naples "Federico II" Naples, Naples, Italy

## Abstract

**Background:**

Liver represents the main organ subject to metastases from colorectal tumors. Resections of liver metastases from colorectal cancer have a well-considered therapeutic role underlined by survival of 5 years by approximately 50-60% of surgical cases as is deduced from an analysis of the most recent literature. The objective of surgery is to eradicate the metastases present and obtain a margin free from neoplastic impact of amplitude of approximately 1 cm with residual liver quantity at the end of the intervention that allows the patient to survive. Currently the dimensions and the number of colorectal liver metastases (CRLM) do not limit the hepatectomy. Purpose of this work is to evaluate the survival, according to our case studies of patients treated only with the wedge resection (atypical resection) approximately 1 cm from the margins of metastases.

**Methods:**

In *"A. Rizzoli" Lacco Ameno Hospital (Ischia)*, from 2005 to 2010, 12 liver resections were performed for metastases from colorectal carcinoma with atypical resection. Synchronous surgical treatment with resection of the colorectal carcinoma and metastases was performed in 6 patients, 2 female and 4 male (Group 1). Surgical liver metastasectomy post-colectomy was performed on 6 patients, 3 female and 3 male (Group 2).

**Results:**

No patient was treated with chemotherapy. The mortality rate of intraoperative and perioperative infection was in both cases of 0%. Survival:11 patients treated surgically from 2005-2010 with synchronous surgery resection (Group 1) and liver metastasectomy (Group 2) are currently living. One 77-years-old patient died three years after surgery for BPCO.

**Conclusions:**

This result was able to be obtained due to the wedge resection technique routinely used in our Hospital, associated with the indispensable use of intraoperatory ultrasound (IOUS). Significant differences between the synchronous and non-synchronous intervention emerged only regarding the number of days of hospital stay, higher in the first case.

## Introduction

Colorectal cancer is third in incidence in the western countries, only preceded of prostate and lung cancer in men and breast and lung cancer in women. The combination of molecular events that lead to colorectal adenocarcinoma is very varied and includes genetic and epigenetic anomalies. Were described at least two different genetic pathways. These are the APC/beta catenine pathway associated with WNT and the classic adenoma-carcinoma sequence (which represents up to 80% of sporadic colon tumors); and the pathway of the micro satellite instability, associated with defects in the system for repairing the DNA mismatch. There is, then, a third group of colon tumors presenting an increase in the methylation of the CpG island without micro satellite instability; many of these tumors hide Kras mutations. Many causes work together to determine the colorectal tumor; among them were identified some associated with lifestyle, others genetic and others of non-hereditary type. Lifestyle and family history have long been identified as factors increasing the risk of occurrence of these lesions: nutritional factors (diet with a large amount of animal fats and refined sugars and low in fiber), obesity, low physical activity, smoking and excess alcohol [1]. There are many hereditary pathologies, such as genetic factors, that predispose onset of colorectal carcinoma, among these the syndromes characterized by the occurrence of polyps (most of the colorectal carcinomas, approximately 80%, originated from precancerous lesions such as adenomatous polyps with dysplastic component), familial adenomatous polyps (FAP), Polyps Associated with MYH gene (MAP), Gardner's syndrome, Turcot's syndrome; and those without polyps such as Lynch's syndrome or Hereditary Non Polyposis Colorectal Carsinosis (HNPCC)[2-4]. The probability that a polyp of the colon may evolve toward an invasive form of cancer depends on the dimensions of the polyp itself: is low (less than 2%) for dimensions lower than 1.5 cm; intermediate (2% - 10%) for dimensions between 1.5 - 2.5 cm; significant (more than 10%) for lesions of dimensions greater than 2.5 cm.[5]. Additional risk conditions that are included among non-hereditary factors consist of chronic inflammatory intestinal illnesses such as Crohn's disease and ulcerous rectal colitis [6]. Age also is among the risk factors; the occurrence is 10 times higher among people between the ages of 60 and 65 compared to those from 40 to 45. Colorectal tumor is located, in half of the cases, in the sigma and in the rectum; in one case in four (25%) of patients, the ascending colon is affected, while the location of the illness in the transverse and descending colon is verified in approximately one case in five (20%). Most frequent istotype of large intestine tumor is represented by adenocarcinoma, in 90-95% of all colon tumors. Metastasis at the hepatic level represents one of the most frequent forms of neoplastic progressions the large intestine, followed by lungs, encephalon and skeleton [7]. It is estimated that the 25% of patients with colorectal carcinoma presenting liver metastases have already been diagnosed with this. Surgical resection of colorectal liver metastases (CRLM) is currently the only therapeutic option able to significantly improve long-term survival, offering possibility of potential cure. The 5-year survival rate in patients treated surgically exceeds 58%, while 10 years of survival exceeds 25% [8]. In contrast, patients who are not candidates for hepatectomy present average survival lower than 12 months and at 5 years the survival rate is equal to 0.9% [9]. In this article, we wish to emphasize the aspect of the average survival of the patients undergoing resection of liver metastases, presenting case studies for patients operated on in our center between the years 2005-2010 and thus demonstrate the major role of surgery in liver metastases from colorectal cancer. First we must identify criteria indicating surgical resection. Our objective surgery is to eradicate all liver metastases and create the conditions for at least a centimeter of healthy tissue is left around the lesion removed according to the "wedge resection" approach. In reality, these limits were discussed by Cady and Kokudo, who in their studies affirm the possibility of holding the margins of resection in healthy tissue also at less than 1 cm, when technically not avoidable, without modifying survival and radicalism of the operation[10]. Current approach indicates that the resection may be performed, as long there remains a sufficient amount of liver that allows to patient survive during regeneration phase [11]. It is seen, from literature, that after the resection, at least two contiguous liver segments must be present. The remaining segments must present a branch of portal vein, a branch of the hepatic artery, and a bile duct. Furthermore, liver remaining must represent 20-25% of total functioning hepatic volume [12]. This hepatic volume is calculated based on preoperatory axial imaging and is called a future liver remnant, FLR, and must be without illnesses as cirrhosis, steatosis and steatopathy associated with chemotherapy (CASH). In the past patients with more than three metastases or with extrahepatic diseases, were not eligible for surgery, today introduction of preoperatory chemotherapy and new chemotherapy drugs as well as the progress of modern surgical technique, amplified the outcome of eligible patients. Currently size and number of liver metastases from colorectal carcinoma (CRLM) do not limit hepatectomy, thus making possible the removal of all metastases present with negative margins, maintaining adequate functionality of remaining liver. As an example we can report a case treated by us in 2009, included in group 2, of a female patient of about 60 years subject to surgical treatment to remove seven liver metastases from colorectal carcinoma. The patient, is in good health, is regularly subject to monitoring and has not presented relapse. The presence of extrahepatic disease does not automatically exclude the patient from surgery. Cases of long-term survival have been reported in a significant number of patients who underwent a complete resection of extrahepatic lesions, especially in the case of pulmonary metastases. Decision-making in these cases is strictly related to the experience of the medical and surgical team, the prognostic clinical factors (performance status, synchronous and metachronous metastases, time to appearance, serological tumour marker levels) and the patient's life expectancy [13]. The optimal surgical strategy for patients with synchronous colorectal liver metastases is not yet well-considered. Traditionally, the standard therapy for most patients with colorectal cancer and synchronous colorectal liver metastases consists of colorectal resection first to prevent bleeding, perforation or obstruction, followed 6 weeks later by staged liver resection [14]. Alternatively patients may be subject simultaneously to colorectal and liver resection. A further therapeutic option for patients with synchronous colorectal liver metastases (CLM) consists of the "liver-first approach"- firstly starting with chemotherapy, secondly doing the liver surgery and lastly, performing the colorectal resection. The rationale of this approach is the reflection that, in patients with advanced or technically doubtful resectable synchronous liver metastases from colorectal cancer, CLM might progress during treatment of the primary, precluding curative treatment in a second stage. Since the curability of liver metastases in this situation but not the primary tumor decides the prognosis of the patient, resection of the primary should be postponed. Initial chemotherapy would control and would reduce the dimensions of liver metastases and would be contextual to radiotherapy for the primary tumor [15]. In our experience, the optimal surgical strategy for the patients that present metastases and primary tumors is the synchronous removal of the primitive tumor and liver metastases. Patients, therefore, may undergo simultaneous colorectal and liver resection. In patients with inoperable liver metastases, neoadjuvant chemotherapy may reduce their capacity, resizing them and thus making the patient a candidate for surgical intervention. According to the literature, the use of Folfox (5-fluorouracil infusion + oxaliplatin), Folfiri (5-fluorouracil, leucovorin, irinotecan) or Folfirinox (oxaliplatino, irinotecan, 5-fluorouracil and leucovorin) combined with biological agents such as cetuximab (better prognosis in the cases of mCRC with Kras wild type), may improve the rates of response and make the 20% of patients considered inoperable, possible candidates for surgical treatment [16]. Standard chemotherapies have systemic toxicities and different efficacy [17-19]. Conversely, there is no hint of a remodeling of the Ca^2+ ^toolkit, that has been observed in other malignancies, including renal cellular carcinoma[20,21], and prostate cancer[22], and has been put forward as alternative target for selective molecular therapies [19]. In this cancer main consequences of neoadjuvant chemotherapy, however, may be: liver toxicity, steatosis, complications of the biliary tract and susceptibility to infection. To minimize the complications, surgical intervention should be performed approximately one month after the end of chemotherapy.

### Materials and methods

Purpose of this work is to evaluate survival, regarding our case studies of all patients under our observation and treated only with the surgical technique of wedge resection (atypical resection) in order to obtain a margin of at least 1 cm free from metastatic involvement. This includes evaluating mortality differences and in peri- and post-operatory complications among the patients undergoing synchronous and non-synchronous resection interventions. Liver resection is considered typical when it is conducted on an anatomic fissure plan architecturally distinct in the vascular structures, especially based on the portal system. In contrast, liver resection is considered atypical, the extension of the pathological process is considered rather than the vascular architecture of the liver.

In "A. Rizzoli" Lacco Ameno Hospital (Ischia), from 2005 to 2010, 12 liver resections were performed for colorectal carcinoma metastases with atypical resection. Patients were retrospectively divided in two groups:

Group 1: six patients, two female and four male, subject to synchronous surgical treatment with resection of the colorectal carcinoma and metastases;

Group 2: six patients, three female and three male, subject to surgical treatment of post-colectomy liver metastasectomy.

An interesting fact is that none of the patients in our study was subjected to preoperative or postoperative chemotherapy. The average age of the patients was 60 years (range 43 - 77), with prevalence for men (7 cases compared with 5), but this is considering the fact that the colorectal neoplasias are most frequent in these latter. Selection criteria for our patients were the following:

1. General operability of the patient in consideration of concomitant diseases;

2. Adequate FLR (future liver remnant);

3. At least two remaining contiguous hepatic sectors after the resection that present adequate blood flow [23]. The clinical risk score (CRS) was useful to select eligible patients and estimate an initial prognostic preoperatory value. It assigns one point to each of the following criteria:

1. Lymph nodes condition;

2. Disease-free interval from the primary to discovery of the liver metastases of < 12 months;

3. Number of tumors > 1;

4. Preoperative carcinoembryonic antigen (CEA) level > 200 ng/ml;

5. Size of the largest tumor > 5 cm.

The 5 year survival rate for patients with 0 points was 60%, whereas that for patients with 5 points

was 14%. Fong et al. concluded that patients with a CRS of 0, 1, 2, have a highly favorable outcome and surgical resection is undoubtedly rational therapy for such patients. Patients with CRS of 3 or 4 have worst prognosis and they should be preventively treated with adjuvant therapy [24].

### Liver anatomy

**Figure 1:**
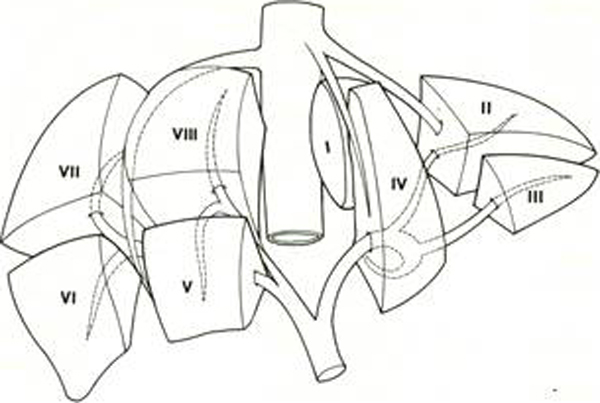
Segmentazione epatica by Couinaud.

Liver anatomy was not widely understood until the middle twentieth century. In the most anatomical text-books, the liver appears little more than a box into which vessels enter and leave but without any internal anatomy. Claude Couinaud et al in France first described the segmental anatomy of the liver, which is essential to modern liver surgery. In substance the liver has 4 sectors and 8 segments. Every segment has a portal vein, hepatic artery, and bile duct and hepatic vein branches. This means they can all be resected separately leaving for the most part the other segments of the liver uncompromised [25]. Even today liver segmentation is inspired by Couinaud and divides the liver into 8 segments, each independent regarding the arterial and venous contribution as well as the vein flow (Figure 1). Main fissure demarcates the line between the gallbladder and lower caval vein and the plane of the medial-suprahepatic vein. Spigelian caudate lobe is placed dorsally and represents segment I; Left lobe is divided into segments II, III, IV; Right lobe in the segments V, VI, VII, VIII. Suprahepatic veins delimit the internal segments of the liver:Right suprahepatic veins separates the VI and the VII segment from V and from VIII; Medial suprahepatic veins separates the V and the VIII segment from IV.

### Preoperative assessment

Medical history conducted in patients with neoplastic disease showed weight loss, low-grade fever and pain. Laboratory exams showed leukopenia, increased transaminases, alkaline phosphatase, as well as hyperbilirubinemia and hypoalbuminemia. Instrumental methods used were the ultrasound, MRI, CT scan with cytology and sometimes angiography. Furthermore, in all patients was observed an increase in some tumoral markers, including CEA, alpha-fetoprotein and TPA.

### Surgical procedure

**Figure 2:**
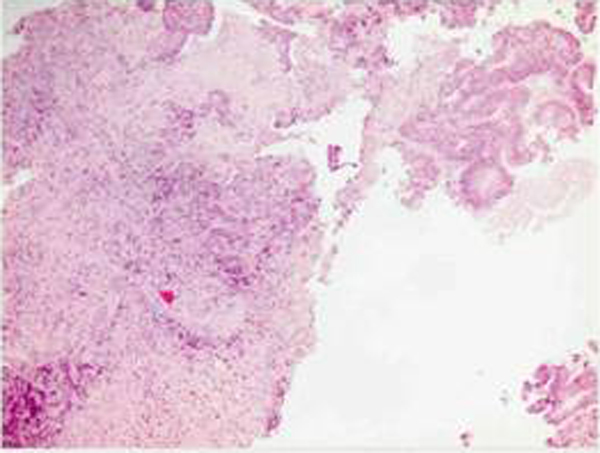
**Biopsy of the case with a diagnosis of hepatic infiltration colonic adenocarcinoma**.

**Figure 3:**
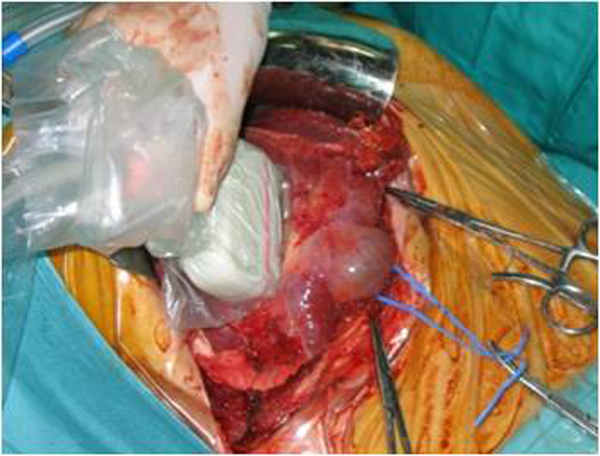
*Intraoperatory ultrasound*: the intraoperatory high resolution ultrasound may identify very small liver lesions (including the marginal ones) that are often unrecognized preoperatory with other imaging methods.

Now we illustrate the main points of the phases of surgical resection of liver metastases in a patient of 52 years, treated in our hospital in 2006 (Group 2) (Figure 2 and 3). In 2004, Sahani et al. reported advantages of the IOUS hepatospecific regarding the MRI with contrast media. The sensitivity of IOUS and MRI was respectively 94.3% and 86.7% [26].

### Multiple liver metastases (Group2)

**Figure 4:**
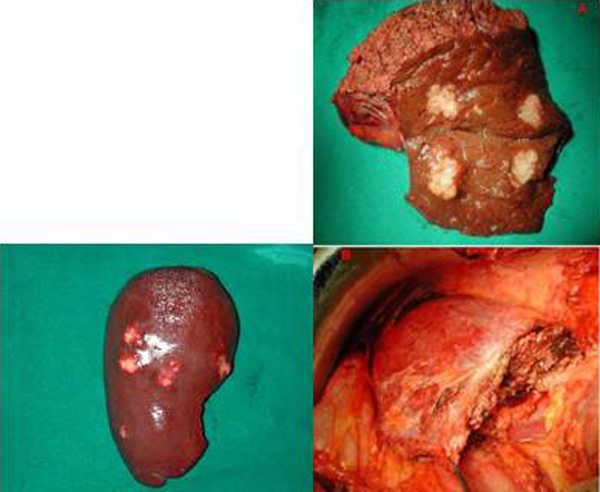
Resection margins of seven hepatic metastases < 1 cm from the lesion (Group 2).

Other interesting element emerged evaluating the patient 60 years already described, belonging to group 2, wich were removed well seven liver metastases. The important aspect is determined by the mini-invasive surgical treatment of hepatic metastasis because the resection was performed with margins from 0.5 cm to 1 cm., under strict control intraoperative ultrasound and documented in various phases (Figure 4). The patient is alive today and has no metastatic relapse in routine follow-up.

### Liver metastasis and colorectal carcinoma (synchronous surgery resection) (Group1)

**Figure 5:**
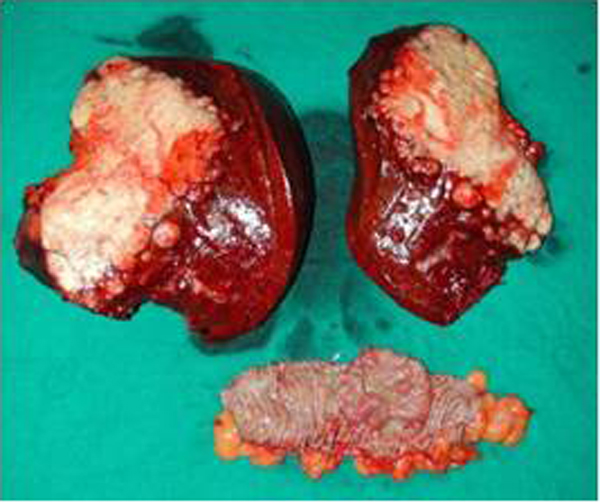
Surgical sample of liver metastasis and colon carcinoma (synchronous surgery resection) Group1.

**Figure 6:**
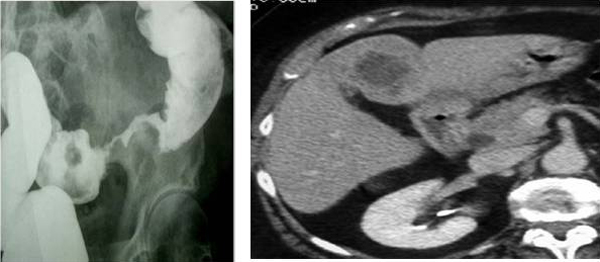
Hemicolectomy sin + metastasis of 6x4 and 7x5 cm (Group1).

We illustrate the case of a patient of 67 years operated in our hospital in 2005, belonging to group1, submitted to surgical treatment of simultaneous resection of adenocarcinoma of the ascending colon and liver metastasis. The patient is currently undergoing periodic monitoring (Figure 5). Finally we examine the case of another patient 71 years of age treated by us in 2007, which is also subjected to surgical treatment of synchronous resection of adenocarcinoma of the left colon and liver metastastasi two large 6x4 and 7x5 cm. (Group1) (Figure 6).

## Results

Twelve patients affected by colorectal carcinoma and liver metastasis were treated surgically at the Hospital in Ischia through:

1) Synchronous surgical treatment with resection of colorectal carcinoma and metastasis, performed in 6 patients, 2 female and 4 male (Group 1).

2) Surgical treatment of liver metastasectomy post-colectomy performed in 6 patients, of which 3 female and 3 male (Group 2).

### Mortality

The mortality rate of intraoperative and perioperative infection was in both cases of 0%. ***Postoperatory complications***

A patient treated in our hospital of liver metastasis (Group 2), with a broncopnemopatia chronic obstructive (BPCO) history, reported a right pleural effusion after six days, he was quickly treated. This 77 years-old patient died three years after intervention for BPCO. Another female patient, 65 years of age with metastasis in the liver segments VII and VIII infiltrating the diaphragm, pneumothorax reported in the sixth day, treated with aspiration and medication (Group 2).

### Hospital stay

**Figure 7:**
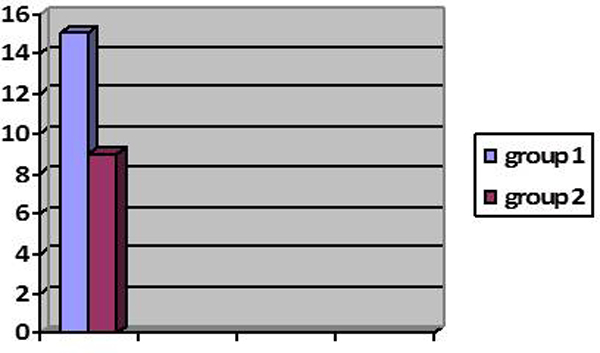
**Average hospital stay**. Synchronous resection of colorectal liver metastases, 15 days (Group 1). Resection of liver metastases, 9 days (Group 2).

The average hospital stay is of 15 days after the synchronous surgical treatment of colorectal carcinoma and liver metastasis (Group 1), while the average stay is 9 days after the liver metastasis (Group 2) (Figure 7).

### Survival

Eleven patients treated surgically from 2005-2010 with synchronous surgery resection (Group 1) and liver metastasectomy (Group 2) are currently living. One 77-years-old patient died three years after surgery for BPCO.

### Monitoring hemostasis

In no case was it necessary to perform transfusions on the surgically-treated patients.

## Conclusion

Cancer and other chronic disease have an increased incidence in old age induced by oxidative stress mechanisms [27,28]. Oxidant overproduction occurs in response to several stressors like drugs, chemicals, hypercaloric diet and physical exercise [29]. Colorectal cancer is third in incidence in the western countries and median age of occurrence is in the sixth and seventh decades. Surgery for colorectal liver metastasis is now well established, it has produced very significant benefits for patients. According to our experience, it is useful to surgically treat all patients with liver metastasis removables and you need to resect as much as possible metastases as long as you manage to get a free margin of at least 1 cm without neoplastic disease. Outcome was obtained in 11 patients treated with the exception of female patient, 60 years of Group 2, who had seven liver metastasis removed, leaving, however, a free margin from the lesion equal to 0.5 cm of free tissue. This result was able to be obtained, in our opinion, due to the wedge resection technique routinely used in our center, associated with the indispensable use of intraoperatory ultrasound (IOUS). The confirmation of the resectionability of the secondary effects on the liver and the safety of the surgical strategy to adopt, including the delimitation of the resection margin, only takes place on the operating table after bimanual palpation and intraoperatory ultrasound. This may in fact indicate foci of the disease smaller than 3 mm not shown in the investigations, evaluate the anatomic relationship between the tumor and the vascular peduncle and guide the excision of lesions. The occurrence of operatory mortality and infection was equal to 0% for both groups. No significant difference emerged in mortality between patients subject to synchronous and non-synchronous resection; The times of stay regarding synchronous intervention were higher compared with metastasectomy. Since 2011 we have surgically treated other cases that we do not include in our work for brief follow-up.

## Competing interests

The authors declare that they have no competing interests.

## Authors' contributions

DL: conceived the study, carried out the surgical procedures, analyzed and interpreted the data. AM: conceived the study, carried out the surgical procedures, analyzed and interpreted the data. SC: critically revised the manuscript. NAC: performed statistical analysis. AR: analyzed and interpreted the data. MV: critically revised the manuscript. MR: perfomed image diagnostic procedures, critically revised the manuscript. EAG: perfomed image diagnostic procedures, critically revised the manuscript. BA: analyzed and interpreted the data, critically revised the drafted manuscript. MPC: conceived the study, analyzed and interpreted the data, drafted the manuscript. All authors read and approved the final manuscript.

## Authors' information

DL: Staff Surgeon at *Lacco Ameno Hospital (Ischia)*

AM: Head Surgeon at *Lacco Ameno Hospital (Ischia)*

SC: Vascular Perfusionist

NAC: Economist for Health

AR: Student at University of Naples "Federico II"

MV: Associate Professor of Endocrinology at University of Salerno

MR: Associate Professor of Radiology at University of Rome "Sapienza"

EAG: Associate Professor of Radiology at University of Cagliari

BA: Associate Professor of Surgery, University of Naples "Federico"

MPC: Assistant Professor of Anatomy, University of Naples "Federico"

## Declarations

Publication charges for this article were covered by research funds of the project Bando Faro 2012 - Finanziamenti per l'Avvio di Ricerche Originali, cofounded by the Compagnia di San Paolo and by the Polo per le Scienze e le Tecnologie per la Vita of the University Federico II in Naples.

This article has been published as part of *BMC Surgery *Volume 13 Supplement 2, 2013: Proceedings from the 26th National Congress of the Italian Society of Geriatric Surgery. The full contents of the supplement are available online at http://www.biomedcentral.com/bmcsurg/supplements/13/S2
